# Genome assembly of *Medicago truncatula* accession SA27063 provides insight into spring black stem and leaf spot disease resistance

**DOI:** 10.1186/s12864-024-10112-9

**Published:** 2024-02-23

**Authors:** Jacob R. Botkin, Andrew D. Farmer, Nevin D. Young, Shaun J. Curtin

**Affiliations:** 1 United States Department of Agriculture, Plant Science Research Unit, St Paul, MN 55108 USA; 2 Department of Plant Pathology, University of Minnesota, St. Paul, MN 55108 USA; 3 Department of Agronomy and Plant Genetics, University of Minnesota, St. Paul, MN 55108 USA; 4 Center for Plant Precision Genomics, University of Minnesota, St. Paul, MN 55108 USA; 5 Center for Genome Engineering, University of Minnesota, St. Paul, MN 55108 USA; 6 National Center for Genome Resources, Santa Fe, NM 87505 USA

**Keywords:** Genome assembly, *Medicago truncatula*, Spring black stem and leaf spot disease, Necrotroph, Disease resistance

## Abstract

*Medicago truncatula*, model legume and alfalfa relative, has served as an essential resource for advancing our understanding of legume physiology, functional genetics, and crop improvement traits. Necrotrophic fungus, *Ascochyta medicaginicola*, the causal agent of spring black stem (SBS) and leaf spot is a devasting foliar disease of alfalfa affecting stand survival, yield, and forage quality. Host resistance to SBS disease is poorly understood, and control methods rely on cultural practices. Resistance has been observed in *M. truncatula* accession SA27063 (HM078) with two recessively inherited quantitative-trait loci (QTL), *rnpm1* and *rnpm2,* previously reported*.* To shed light on host resistance, we carried out a *de novo* genome assembly of HM078. The genome, referred to as *MtHM078 v1.0*, is comprised of 23 contigs totaling 481.19 Mbp. Notably, this assembly contains a substantial amount of novel centromere-related repeat sequences due to deep long-read sequencing. Genome annotation resulted in 98.4% of BUSCO fabales proteins being complete. The assembly enabled sequence-level analysis of *rnpm1* and *rnpm2* for gene content, synteny, and structural variation between SBS-resistant accession SA27063 (HM078) and SBS-susceptible accession A17 (HM101). Fourteen candidate genes were identified, and some have been implicated in resistance to necrotrophic fungi. Especially interesting candidates include loss-of-function events in HM078 because they fit the inverse gene-for-gene model, where resistance is recessively inherited. In *rnpm1*, these include a loss-of-function in a disease resistance gene due to a premature stop codon, and a 10.85 kbp retrotransposon-like insertion disrupting a ubiquitin conjugating E2. In *rnpm2*, we identified a frameshift mutation causing a loss-of-function in a glycosidase, as well as a missense and frameshift mutation altering an F-box family protein. This study generated a high-quality genome of HM078 and has identified promising candidates, that once validated, could be further studied in alfalfa to enhance disease resistance.

**Supplementary Information:**

The online version contains supplementary material available at 10.1186/s12864-024-10112-9.

## Introduction

The sequencing of the model legume *Medicago truncatula* has delivered substantial insights into legume biology, in particular symbiotic nitrogen fixation studies. *M. truncatula* has been utilized as a model legume due to qualities which include having a relatively small genome, self-fertility, a short generation time, its symbiosis with rhizobia, ease of transformation, and being a diploid relative of forage crop *M. sativa* (alfalfa). The *Mt4.0* reference genome of *M. truncatula* accession A17 was generated using the whole-genome shotgun approach with Illumina, 454, and Sanger DNA sequence reads [1]. The *Mt4.0* genome is 390 Mbp with 37,561 functionally annotated gene models. Later, 109x coverage of Pacific Biosciences (PacBio) long-read DNA sequences was used to further improve the reference genome. The *Mt5.0* version reported a genome size of 429.43 Mbp, representing the majority of the 465 Mbp estimated haploid genome size of *M. truncatula* [1, 2].

In the last decade, researchers generated significant increases in genomic resources for *M. truncatula*. For instance, Stanton-Geddes et al. [3] identified single nucleotide polymorphisms (SNPs) in 288 *Medicago* accessions from the Medicago HapMap diversity collection based on Illumina sequencing. Within this collection, *de novo* assemblies were generated with 120x coverage of Illumina sequence data for 15 accessions, including HM004, HM010, HM022, HM023, HM034, HM050, HM056, HM058, HM060, HM095, HM101 (A17), HM125, HM129, HM185, and HM340 (R108) to investigate the pan-genome [4]. While these assemblies provided information about the pangenome, the fragmentation observed in short read assemblies complicates synteny analysis over large genomic regions.

Recently, long-read sequencing produced a high-quality genome assembly of the of *M. truncatula* ssp*. tricycla* accession R108, the transformable line for *M. truncatula*. In the *R108 v1.0* genome, 399 Mbp was assembled from 100x coverage of PacBio long-read DNA sequences using Falcon v.0.4 [5]. After Hi-C anchoring, 39,027 protein-coding gene models were identified, of which 36,994 genes shared homology with annotated plant proteins in TAIR, Phytozome, Swissprot, and RefSeq databases. A comparative analysis between the *Mt5.0* and *MedtrR108_hic* genome revealed a high degree of collinearity across chromosomes 1-8, except for a 12 Mbp and 17 Mbp translocation between chromosomes 4 and 8 in A17 [6]. Overall, PacBio long-read DNA sequence data has been a determining factor in producing reference-quality genomes of *M. truncatula*.

Legume yields are reduced by an array of fungal diseases. Ascochyta blights are destructive foliar diseases of legumes, which are caused by necrotrophic fungi in the *Didymella, Phoma,* and *Ascochyta* genera. The *Ascochyta* reside in the Dothideomycetes class and Pleosporales order, which includes many phytopathogenic fungi that produce host-selective toxins (HSTs) [7]. Spring black stem and leaf spot (SBS) disease is an important disease of *M. sativa* (alfalfa) and its diploid relative *M. truncatula* [8]. This disease is globally distributed, but is one of the most severe foliar diseases of alfalfa in Canada, Iran, Australia, and Europe [8–11]. *Ascochyta medicaginicola*, previously known as *Phoma medicaginis*, is the causal agent of SBS disease. Research into the interactions between *M. truncatula* and *A. medicaginicola* offer the potential to elucidate host response mechanisms to necrotrophic fungal pathogens. Symptoms of SBS disease include necrotic black lesions with chlorosis in surrounding areas on vegetative tissues. As the disease progresses the lower canopy is defoliated, and pycnidia develop on the dead foliage. Pycnidia are asexual fruiting bodies which produce primary inoculum in future growing seasons. To manage SBS disease moderately resistant cultivars and disease-free seed are planted, infected fields are cut early to minimize losses, and crop residue is managed by tilling and grazing to reduce primary inoculum in future growing seasons.

In the SBS pathosystem complete host resistance has not been observed. For *A. medicaginicola*, spore germination, fungal penetration, and pycnidia development were delayed by 24 hours for resistant alfalfa genotypes compared to susceptible ones [9]. Eighty-six *M. truncatula* accessions from the South Australian Research and Development Institute (SARDI) collection were screened for resistance to SBS disease, and while most accessions were susceptible, genotype-specific resistance was observed for 16 accessions, including accession SA27063, referred to by the Medicago HapMap identifier HM078 [10]. For accession SA27063 (HM078), the mean disease rating was 1.64 on a 1-5 disease reaction scale increasing in severity against *A. medicaginicola* isolate OMT5. The development of pycnidia on senescing leaves was delayed by 2-3 weeks, and HR-like spotting was observed. SA27063 (HM078) was originally collected by the French Institut National de la Recherche Agronomique (INRA) from Greece (38.083°N, 22.570°E). It was later utilized as the resistant parent in a QTL mapping study to explore the basis for its resistance to SBS disease. Two alternate recessive QTL, *rnpm1* and *rnpm2*, that explained 33.6% and 29.6% of phenotypic variance for SBS disease were identified with *P* < 0.00001 and an LOD of 7.37 and 6.77, respectively [12]. QTL *rnpm1* was mapped to the upper arm of linkage group 4 in an F_2_ population derived from SA27063 (HM078) and A17 (HM101), and is linked to a cluster of Toll/Interleukin1 receptor-nucleotide binding site-leucine-rich repeat (TIR-NBS-LRR) genes. Fine mapping was not performed for *rnpm1* because of low viability of this population’s progeny, which was determined to be caused by the chromosome 4-8 translocation in A17 [13]. QTL *rnpm2* was mapped to linkage group 8 in an F_2_ population derived from SA27063 (HM078) and SA3054, and was fine mapped by recombinant breakpoint analysis in F_3_ families to a ~480 kbp region [12]. The fine mapping of *rnpm2* provides a higher level of confidence that this locus segregates with SBS disease resistance.

In this study, we leveraged advances in sequencing and comparative genomics to offer insight into the basis of resistance to SBS disease. To accomplish this, we conducted a *de novo* genome assembly of *M. truncatula* accession SA27063 (HM078) from PacBio long-read DNA sequence data, referred to as the *MtHM078 v1.0* genome. Protein-coding genes were annotated with the BRAKER/TSEBRA pipeline. Previously described QTL regions, *rnpm1* and *rnpm2*, for SBS disease resistance were investigated for synteny, structural variation, and missense SNPs across multiple *M. truncatula* genomes, including the reference-quality genomes of SBS-susceptible accessions A17 (HM101) and R108 (HM340), as well as SBS-resistant accession SA27063 (HM078). Candidate genes for disease resistance were identified for future validation.

## Material and methods

### Sequence data accessed

Sequence data was accessed from a variety of sources. The data includes *M. truncatula* genome sequences, genotypic data, gene family classifications, and a transcriptomic database. The *M. truncatula* genomes accessed include the *Mt5.0* reference genome of accession A17, the *MedtrR108_hic* genome of accession R108, and numerous other accessions available on Legume Information System (LIS) [14]. Descriptions and links for the data accessed are provided (Additional file  2: Table S1).

### Plant growth conditions

Germplasm of *M. truncatula* accessions SA27063 (HM078), A17 (HM101), and R108 (HM340) was obtained from the Medicago HapMap germplasm collection at the University of Minnesota, St. Paul. Seed was scarified by treating with 2 mL of concentrated sulfuric acid for 7 minutes in a 15 mL conical tube, followed by 3 successive washes with sterile de-ionized (DI) water. Seedlings were grown in autoclaved potting soil (Sun Gro Professional Growing Mix, Sun Gro Horticulture, Agawam, MA, USA) with Osmocote Plus fertilizer (Scotts Miracle-Gro, Marysville, OH, USA) in a growth chamber at 22-24°C with 16 hours of light per day.

### Disease scoring data accessed

SBS disease ratings for *M. truncatula* accessions A17 (HM101), SA28064 (HM002), and SA27063 (HM078) were obtained from Ellwood et al. [10]. Disease ratings were recorded on a 1-5 scale increasing in severity where accessions with a disease rating of 2 or below are considered resistant and accessions with a disease rating of 3 and above are susceptible. *M. truncatula* accessions SA28064 (HM002, 1.33) and SA27063 (HM078, 1.64) were resistant, while A17 (HM101, 4.15) was susceptible when challenged with *A. medicaginicola* isolate OMT5. In this study, *M. truncatula* SA27063 (HM078), A17 (HM101), and R108 (HM340) were inoculated with *A. medicaginicola* isolate OMT5 using the spray inoculation methods described in Ellwood et al. [10] to validate resistance and susceptibility.

### Nuclei isolation

Immature leaf tissue from *M. truncatula* HM078 was harvested from 3-week-old plants, rinsed with sterile DI water with 50 ppm Tween®20 surfactant (Sigma-Aldrich, St. Louis, MO), and subjected to the Nuclei Isolation LN2 Plant Tissue Protocol (Circulomics, Baltimore, MD, USA) prior to DNA extraction as described by Dvorianinova et al. [15]. Briefly, 2 grams of tissue was subjected to grinding in liquid nitrogen for 20 minutes. Ten milliliters of the ice-cold nuclei isolation buffer was added to frozen ground tissue immediately in a 50 mL conical tube. A Tube Revolver (Thermo Fisher Scientific, Waltham, MA, USA) was used to mix the tissue suspension at 15 rpm for 15 minutes. The lysate was filtered using a 50 mL Steriflip (Millipore Sigma, Burlington, MA) with a 20 µm pore size, and centrifuged at 3,000 x g for 20 minutes at 4°C. The supernatant was decanted, and the pellet was resuspended in 15 mL of ice-cold nuclei isolation buffer and centrifuged at 3,000 x g for 10 minutes. The rest of the protocol was followed according to the manufacturer’s specifications resulting in a 1 mL nuclei suspension.

### High molecular weight DNA extraction and sequencing

A high molecular weight (HMW) DNA extraction was carried out for HM078 using the Monarch HMW DNA Extraction Kit for Cells & Blood (New England Biolabs, Ipswich, MA) following the kit’s protocol for cells. The Short Read Eliminator Kit (Circulomics Inc., Baltimore, MD, USA) was used to deplete DNA fragments below 25 kbp. The resulting HMW DNA was quantified with a Qubit 3.0 Fluorometer (Thermo Fisher Scientific, Waltham, MA, USA), and approximately 200 ng was run on a 0.8% agarose gel along with the NEB Quick-Load 1 kbp Extend DNA ladder (New England Biolabs, Ipswich, MA). Pulse-field capillary electrophoresis (FEMTO-Pulse, Agilent Technologies Inc., Santa Clara, CA, USA) was utilized to assess the relative quantity of HMW DNA at various fragment lengths. HMW DNA was stored at 4°C until library preparation.

PacBio long-read DNA sequencing was conducted at the University of Minnesota, Twin Cities, Genomics Center. Prior to library preparation, HMW DNA was purified with AMPure PB beads (Pacific Biosciences, Menlo Park, CA, USA). The PacBio SMRTbell prep kit 3.0 (Pacific Biosciences, Menlo Park, CA, USA) was used to for library preparation following the manufacturer’s specifications, and long-read sequencing was performed on the Sequel II system with two 8M SMRT Cells running for a 30-hour period (Pacific Biosciences, Menlo Park, CA, USA).

### RNA extraction and sequencing

A total of 12 tissue samples consisting of immature leaf (1), apical meristem (1), root (1), and mature leaf (9) tissue were harvested from 4-week-old *M. truncatula* accession SA27063 (HM078) and subjected to RNA extraction using the Qiagen RNeasy Mini Kit for Plants (Qiagen Inc., Valencia, CA, USA) following the manufacturer’s protocol. Tissue samples were collected from plants grown in the plant growth conditions described above and not exposed to any treatment. Mature leaf tissues were collected from separate biological replicates. The quantity of extracted RNA was measured using a NanoDrop 2000 Spectrometer (Thermo Fisher Scientific, Waltham, MA, USA) and fragment degradation was initially assessed using the Qubit 3.0 Fluorometer RNA IQ Assay (Thermo Fisher Scientific, Waltham, MA, USA). Illumina RNA sequencing was conducted at the University of Minnesota, Twin Cities, Genomics center. Fifteen TruSeq unique dual-indexed (UDI) stranded mRNA libraries were prepared, combined in a single pool, and sequenced on a single lane of NovaSeq S4 2x150-bp flow cell.

### *De novo* genome assembly and scaffolding

PacBio sequencing adapters were removed from PacBio HiFi CCS long-reads using HiFiAdapterFilt v2.0.1 [16]. *De novo* genome assembly was performed using HiFiasm v0.16.0 [17] using default options. The Funannotate v1.8.1 script ‘clean’ was utilized to remove duplicated contigs [18]. BUSCO v5.3.2 [19] was run to evaluate genome completeness by the presence of BUSCO genes using the fabales_od10 dataset (*n*=5,366). *In silico* scaffolding was performed with RagTag v2.1.0 [20] to orient contigs based on a reference genome. The Hi-C scaffolded chromosome-level genome of *M. truncatula* accession R108, referred to as the *MedtrR108_hic* genome [6], was chosen as the scaffolding reference because it lacks the chromosome 4-8 translocation present in *M. truncatula* accession A17 [13]. The scaffolded assembly of *M. truncatula* HM078 will be referred to as the *MtHM078 v1.0* genome. ChromoMap v4.1.1 [21] was used to visualize contig orientation during scaffolding.

### Macro-synteny between chromosome-level genomes of *M. truncatula*

To evaluate macro-synteny between chromosome-level genomes of *M. truncatula* (*Mt5.0*, *MedtrR108_hic*, and the *MtHM078 v1.0* genome) we used tools such as SyRI v1.6.3 [22] and dot plots. For both, whole-genome DNA alignments were produced between the three genomes using Minimap2 v2.1 [23]. For dot plots, the alignments were plotted using ‘pafCoordsDotPlotly.R’ (https://github.com/tpoorten/dotPlotly). For SyRI, whole-genome alignments were plotted using ‘plotsr’ (https://github.com/schneebergerlab/plotsr).

### SNP dendrogram for *M. truncatula* accessions with genome assemblies

We generated a dendrogram based on SNP data for *M. truncatula* accessions with genome assemblies available to approximate genetic relationships between accessions with genomic resources. SNP data for the Medicago HapMap collection was accessed and filtered for accessions with genome assemblies available on LIS (Additional file 2: Table S1). PLINK v1.9 [24] was used to randomly select 50,000 SNPs from the genotypic data, and a dendrogram was generated using SNPRelate v1.28.0 [25] default parameters, and visualized with ggtree v3.2.1 [26].

### Repetitive elements

Prior to genome annotation, repetitive elements were soft masked. RepeatModeler v2.0.1 [27] was used to *de novo* annotate the repetitive element space within the *MtHM078 v1.0* genome. Briefly, transposable elements (TEs) such as long interspersed nuclear elements (LINEs), DNA transposons, long terminal repeat retrotransposons (LTR-RTs), simple repeats, and low complexity repeats were identified. Repetitive elements were classified with RepeatMasker v4.1.1 [28]. The repeat library used for classification was augmented with a *M. truncatula* repeat library downloaded from PlantRep [29].

Centromeric and pericentromeric repeat regions were analyzed based on the larger chromosome representations observed in the *MtHM078 v1.0* genome assembly. Pericentromeric, *Mt*R1 and *Mt*R2, and centromeric, *Mt*R3, repeat probes designed by Kulikova et al. [30] for fluorescent in situ hybridization (FISH) of *M. truncatula* A17 chromosomes were utilized to assess the presence of centromeric repeats computationally [30]. NCBI BLAST+ v2.8.1 [31] was used to align nucleotide sequences of FISH probes to the *MtHM078 v1.0* genome, as well as the *Mt5.0* and *MedtrR108_hic* genome. BLASTn results were filtered based on a nucleotide sequence identity above 98% across 90% of the probe length or greater, a e-value below 1e-05, and a bitscore above 50, and the results were visualized using ChromoMap v4.1.1 [21].

### Long-read coverage in centromeric and pericentromeric regions

We assessed read coverage of PacBio CCS HiFi long-reads in regions containing centromeric and pericentromeric repeats to validate the assembly of these repetitive regions. First, genomic regions homologous to centromeric and pericentromeric repeats were extracted from chromosomes 1-8 using Samtools v1.9 [32] ‘faidx’. Then, long-reads were mapped to these regions using BWA v0.7.17 [33]. Alignment files were indexed and sorted using Samtools v1.9 [32]. Finally, Samtools v1.9 [32] ‘depth’ was used to calculate the coverage of long-reads (>15 kbp) with high-quality alignments (Q>30) at each genomic position, and the results were visualized using R v4.1.2 in R studio v1.4.1717 [34].

### Genome annotation

RNA sequence reads were processed in a series of steps. Cutadadpt v1.18 [35] and was employed to trim Illumina sequencing adapters, and retain RNA sequence reads with Phred-scaled quality scores greater than 30 and longer than 50 bp. MulitQC v1.14 [36] was used to summarize FastQC reports. Next, STAR v2.5.3 [37] was used to perform spliced transcript alignments to the *MtHM078 v1.0* genome*.* STAR v2.5.3 was run in ‘--twopassMode Basic’, ‘--sjdbOverhang 149’, and default parameters. RNA sequence mapping statistics were quality checked with MulitQC v1.14. Samtools v1.9 [32] was used to merge and filter alignment files to only include paired RNA sequence reads with unique alignments to the *MtHM078 v1.0* genome.

Next, a structural annotation of protein-coding genes was conducted using BRAKER1 v1.9 [38] and the RNA sequence alignments. First, GeneMark-ET v4.61 [39] initiated a self-training algorithm for *ab initio* gene prediction. The genes predicted by GeneMark-ET v4.61 are evaluated against quality assessments to select a set of high-quality genes to train Augustus v3.2.3 [40] along with a hints file containing intron locations. After training, Augustus v3.2.3 conducts a separate gene prediction pipeline. Next, predicted protein sequences were obtained from *Mt5.0* and *MedtrR108_hic*, and aligned to the *MtHM078 v1.0* genome using GenomeThreader v1.7.1 [41]. Then, BRAKER v2.02 [42] was run using the protein alignments. In the BRAKER v2.02 pipeline GeneMark-EP v4.61 is run first, followed by Augustus v3.2.3 [40].

Transcript selection was performed with TSEBRA v1.1.0 [43], which collates predictions from BRAKER1 and BRAKER2, and selects the highest scoring transcripts for each gene model. To remove false positive predictions, NCBI BLAST+ v2.8.1 [31] was run to align initial *MtHM078 v1.0* predicted proteins to predicted proteins of legume federation gene families in the *Mt5.0* genome. The legume federation gene families are defined as hidden Markov model (HMM) models based on multiple sequence alignments of proteins from annotated legume genomes available on LIS, and represent conserved legume proteins [44]. *MtHM078 v1.0* gene models were retained if their predicted protein sequences had at least 30% percent amino acid sequence identity, a bit score above 50, and an e-value below 1e-05 with predicted proteins of legume federation gene families. Finally, TSEBRA v1.1.0 was run a second time using options ‘filter_single_exon_genes’ and ‘keep’ to remove single exon genes without homology to conserved legume federation proteins. Finally, BUSCO v5.3.2 was run to evaluate the presence of 5,366 universal orthologs from the fabales lineage within the *MtHM078 v1.0* predicted protein sequences.

### Functional annotation

A total of 37,803 gene models were functionally annotated using InterProScan v5.23-62.0 [45], which utilized HMMER v3.2.1 to search predicted proteins against multiple protein databases, including SMART [46], SFLD [47], Gene3D [48], CDD [49], PFAM [50], COILS [51], MobiDBLite [52], SUPERFAMILY [53], HAMAP [54], PANTHER [55], TIGRFAM [56], PRINTS [57], and PROSITEPROFILES [58]. InterProScan v5.23-62.0 [45] was run to generate both InterProScan lookup numbers as well as Gene Ontology (GO) terms. In addition, NCBI BLAST+ v2.8.1 [31] was run to align the *MtHM078 v1.0* proteins against annotated protein entries for *M. truncatula* in the UniProtKB database and putative gene functions were integrated.

### Visualization of TIR-NBS-LRR genes in *M. truncatula* chromosome-level genomes

Due to the cluster of TIR-NBS-LRR genes being present in *rnpm1*, and the fact this gene family can play an important role in plant disease resistance to necrotrophic fungi, we visualized genome-wide TIR-NBS-LRR genes for A17 (HM101), R108 (HM340), and SA27063 (HM078). Genes models included were classified as TIR-NBS-LRR genes according to the legume federation gene family assignments on LIS. These include proteins with the InterPro domains IPR000157 (Toll/interleukin-1 receptor homology (TIR) domain), IPR000767 (Disease resistance protein), and IPR027417 (P-loop containing nucleoside triphosphate hydrolase), IPR001611 (Leucine-rich repeat), IPR003591 (Leucine-rich repeat, typical subtype), IPR011991 (Winged helix-turn-helix DNA-binding domain), as well as the GO terms GO:0000166 (nucleotide binding), GO:0005515 (protein binding), GO:0006952 (defense response), GO:0007165 (signal transduction), GO:0017111 (nucleoside-triphosphatase activity), and GO:0043531 (ADP binding).

### Locating QTL regions in reference genomes of *M. truncatula*

QTL *rnpm1* and *rnpm2* were located computationally in the reference genomes of *M. truncatula* accessions A17 (HM101), R108 (HM340), and SA27063 (HM078). For QTL, *rnpm1*, a single tightly linked marker “AW256637” was described by Kamphuis et al. [12], which can be assayed using the following primer pair (AW256637: *5’-*TTCACCTAATTTCCATCTATACCATCCATGT*-3’*, *5’-TATTTGTTAGCTTTAGTGATCGCTGCTACAC-3’*). The QTL region *rnpm1* was not fine-mapped due to a large amount of non-viable progeny in the mapping population [12]. Due to the lack of fine-mapping for this region, an arbitrary size of 1 Mbp was selected for examination of this QTL region, centered on marker “AW256637”. For QTL *rnpm2*, Kamphuis et al. [12] describes two flanking markers, “h2_16a6a” and “h2_21h11d”, which can be identified by the following primer pairs (h2_16a6a: *5’-CTGCCGCATATTCAGTTCAT-3’, 5’-*GTGGATCGTTGGAGTGTGTG-3’, h2_21h11d: 5’-*TGGTTGTTAGCCATCCGTTT-3’, 5’-CCTCACTGCTCAAAACCACA-3’*). Primer pairs for both QTL regions were provided as input to Cas-OFFinder v2.4.1 [59] to identify QTL locations based on primer alignments. Cas-OFFinder v2.4.1 was designed to examine off-target binding sites for guide-RNA primers but works effectively to align primers to a genome and identify possible binding sites, the number of mismatches, and amplicon size.

### Analysis of Indels affecting genes in QTL regions

Indels (insertions and deletions) causing amino acid changes or loss-of-function in genes within QTL regions *rnpm1* and *rnpm2* were examined between *M. truncatula* accessions A17 (HM101) and SA27063 (HM078). Minimap2 v2.1 [23] was utilized for whole-genome pairwise alignments and SyRI v1.6.3 [22] was implemented to call indels. Structural variants within QTL regions were evaluated with SnpEff v1.9.6 [60] to classify the effect of indels on genes, such as frameshift variants and loss-of-function events. Sequence-level alignments of homologous genes were performed in Geneious Prime v.2021.2.2 (Biomatters, Ltd) to validate structural variants.

### Missense SNP analysis in QTL regions

Missense SNPs were evaluated within QTL *rnpm1* and *rnpm2* regions to examine predicted changes in amino acids that are present in resistant accession SA27063 (HM078) and absent from susceptible accession A17 (HM101). A similar strategy was previously used to compare missense SNPs between resistant and susceptible accessions of *A. thaliana*, which resulted in the identification of a TIR-NBS-NLR protein *LAZ5* that confers resistance to *Tobacco ringspot virus*, and contributes to susceptibility to the *S. sclerotiorum* [61]. While missense SNPs do not create loss-of-function events that align with the recessive inheritance model for these QTL regions, we reasoned that an amino acid change in HM078 homologs could alter protein functionality and contribute to resistance. We retrieved SNP data for the *M. truncatula* HapMap collection from LIS, which had been called based on the *Mt5.0* genome of A17 (Additional file 2: Table S1). We used this available SNP data rather than the SyRI v1.6.3 generated SNP calls because it was produced using a widely accepted variant calling pipeline for SNPs. Then, we extracted SNP calls across QTL *rnpm1* and *rnpm2* for SBS-resistant HM078 and SBS-susceptible A17. We applied SnpEff v1.9.6 [60] to classify missense SNPs. Missense SNPs present in HM078 and absent from A17 were identified. Sequence-level alignments of homologous genes were performed in Geneious Prime v.2021.2.2 (Biomatters, Ltd) to validate missense variants.

### Micro-synteny within QTL regions

Genome context viewer (GCV) [62] was used to evaluate micro-synteny of the QTL *rnpm1* and *rnpm2* across *M. truncatula* genomes on LIS. Gene families are assigned based on a conserved set of legume federation proteins developed by Stai et al. [44], and gene family color assignments are assigned within one genomic region. GCV plots show presence-absence variation (PAV), copy-number variation (CNV), conservation of gene order, and micro-rearrangements. However, GCV is limited by the annotation accuracy of protein-coding genes in each genome. We produced a GCV plot of each QTL region, *rnpm1* and *rnpm2*, and labeled candidate genes for SBS-disease resistance based on analyses conducted in this study.

### Identification of homologous genes between *M. truncatula* assemblies

To identify homologs of candidate genes between A17 and HM078, the predicted protein sequence of interest was aligned to the other accessions predicted proteins using NCBI BLAST+ v2.8.1 [31]. Proteins were selected for further examination if 30% or more of their total length aligned with a sequence identity above 30%, a e-value below 1e-05, and a bitscore above 50 as described by Pearson [63]. Proteins with significant BLASTp alignments were subjected to pairwise-alignment of amino acid sequences using the Needleman-Wunsch algorithm via EMBOSS Needle [64], and the highest amino acid sequence identity was used to identify putative homologs.

### PCR amplification of *PHO2A* locus

PCR amplification of the *PHO2A* locus (MtrunA17_Chr4g0009054) was performed across five *M. truncatula* accessions to validate structural variation present at this locus. Accessions A17 (HM101), R108 (HM340), SA28064 (HM002), DZA058-J (HM050), and SA27063 (HM078) were subjected to CTAB/Chloroform DNA extraction followed by PCR using GoTaq G2 DNA Polymerase (Madison, WI, USA) with thermocycling conditions described by the manufacturer and optimal annealing temperatures for primer pairs. PCR primers were as follows: to amplify the *PHO2A* gene MtPho2A-F (5’- AGGTTATGTCCTCGACCGCTTCC-3’) and MtPho2A-R (5’- GTATTTTTCAGCTAGGTAACCAGA-3’). For accession DZA058-J (HM050), a more specific reverse primer was (5’- GTATTTTTCAGCAAGGTAACCGGG -3’) was used. To amplify the left and right flanking regions of a retrotransposon-like insertion in the *PHO2A* locus of HM078 the primers 78-insertion-L-F (5’-GCATACACCAGTCACCAAGTTGCC-3’) and 78-insertion-L-R (5’-GTCGTGAGTGGCCTTGCCTT-3’), as well as 78-insertion-R-F (5’-ACACTATTGAATTATTAGTCTCACCC -3’) and 78-insertion-R-R (5’-GTATCCATACTATCCAGGGC -3’) were used, respectively. For a positive control, MtACTIN11-F (5’-ACGAGCGTTTCAGATG-3’) and MtACTIN11-R (5’-ACCTCCGATCCAGACA-3’) were used to amplify the MtACTIN11 locus (MtrunA17Chr7g0223901).

## Results

### A highly contiguous *de novo* genome assembly of HM078

**Fig 1:**
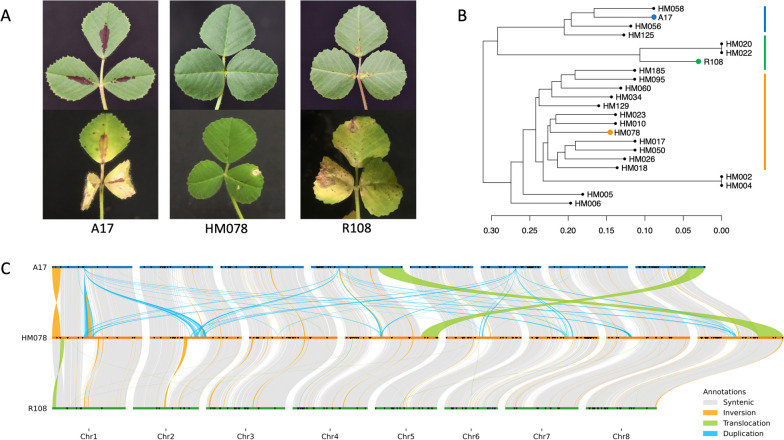
*M. truncatula* accessions A17 (HM101), SA27063 (HM078), and R108 (HM340). **A** Healthy (top) and symptomatic (bottom) leaves for SBS-susceptible A17 and R108 and SBS-resistant HM078 one week post inoculation. **B** SNP dendrogram for *M. truncatula* genotypes with genome assemblies available. **C** Inter-and intrachromosomal whole-genome pairwise alignments identifying structural variation among the chromosome-level genomes of *M. truncatula* for A17 (*Mt5.0*), R108 (*MedtrR108_hic*), and HM078 (*MtHM078 v1.0*). Black triangles indicate TIR-NBS-LRR plant disease resistance genes identified based on legume federation gene families

We sequenced and assembled the genome of *M. truncatula* HM078 to develop a resource for investigating SBS disease resistance. HM078 has resistance to SBS disease resulting in hypersensitive-like spots, whereas A17 and R108 are highly susceptible, with chlorosis, necrosis, and defoliation one week post inoculation (Fig. 1a). We generated a SNP dendrogram that suggests susceptible accessions A17 and R108 are more closely related to each other than to HM078, although Choi et al. [65] proposed they fall in the truncatula and littoralis subclade, respectively, based on plastid phylogenomics (Fig. 1b). High-quality genomic DNA from HM078 was extracted for genome sequencing using the PacBio Sequel II CCS HiFi platform, yielding 5.4 million HiFi reads, or 65.04 Gbp (≥ Q30) that provided 142x fold coverage of the estimated genome size. HiFiasm v0.16.0 assembly generated 266 contigs ranging from 10.8 kbp to 62.1 Mbp and totaling 494 Mbp (contig N50:31.41 Mbp). A total of 99.0% of fabales BUSCOs (*n*=5,366) were present and complete, which was comparable to the reference genomes of A17 and R108 (Table 1). In fact, the *MtHM078 v1.0* genome is missing 37 BUSCOs, while the *Mt5.0* genome is missing 36 and the *MedtrR108_hic* genome is missing 43. Of the missing BUSCOs, *MtHM078 v1.0* and *Mt5.0* are missing 32 of the same BUSCOs, while *MtHM078 v1.0* and *MedtrR108_hic* are missing 29 of the same BUSCOs. Mitochondrial (361,468 bp) and plastid (123,737 bp) genomes were assembled and contained 45.34% and 33.98% GC content, respectively.

**Table 1:** Summary metrics for the *MtHM078 v1.0*, *Mt5.0*, and *MedtrR108_hic* genomes

	***MtHM078 v1.0* (HM078)**	***Mt5.0* (A17)**	***MedtrR108_hic* (R108)**
Total length (Mbp)	494.46	429.43	401.006
Number of contigs	266	62	1005
Contig N50 (Mbp)	31.41	23.3	5.93
Contig L50	6	7	18
Number of scaffolds	10	42	801
Scaffold N50 (Mbp)	62.41	56.24	51.86
Scaffold L50	4	4	4
Longest scaffold (Mbp)	66.46	64.76	55.9
Percent gaps (%)	0.0003	0.13	0.413
GC content (%)	34.48	33	33
BUSCO fabales dataset (genome)	C:99.0%[S:96.3%,D:2.7%],F:0.4%,M:0.6%,n:5366	C:98.9%[S:96.2%,D:2.7%],F:0.4%,M:0.7%,n:5366	C:98.9%[S:95.8%,D:3.1%],F:0.3%,M:0.8%,n:5366

BUSCO abbreviations are *C* Complete, *S* Complete and single-copy, *D* Complete and duplicated, *F* Fragmented, *M* Missing, *N* total BUSCOs analyzed

DNA-based alignments of the nine largest HM078 contigs and the genomes of A17 and R018 showed that HM078 lacked the chromosome 4-8 translocation present in A17 (Additional file 1: Figure S1). Therefore, the *MedtrR108_hic* genome was chosen as a scaffolding reference. Twenty-three contigs ranging in size from 0.06-62.17 Mbp and totaling to 481.19 Mbp were oriented into eight pseudo-chromosomes using RagTag v2.1.0 to produce the *MtHM078 v1.0* genome. An additional 229 small contigs (10-361 kb), consisting of 12.8 Mb, did not anchor and were retained as additional contigs. Notably, representations of chromosomes 2, 6, 7, and 8 were composed of only two contigs, with contig 1 and 2 representing near-complete assemblies of chromosome 4 and 8, respectively (Additional file 1: Figure S2, Additional file 2: Table S2). A whole genome pairwise alignment between *Mt5.0* and *MtHM078 v1.0* using SyRI v1.6.3 identified a total of 1,425,308 SNPs, 151,313 insertions (3.69 Mbp), 144,565 deletions (3.77 Mbp), 4,424 inversions, and 4,887 duplications.

### Repetitive element analysis identifies centromeric sequence in HM078 pseudo-chromosomes

**Fig 2:**
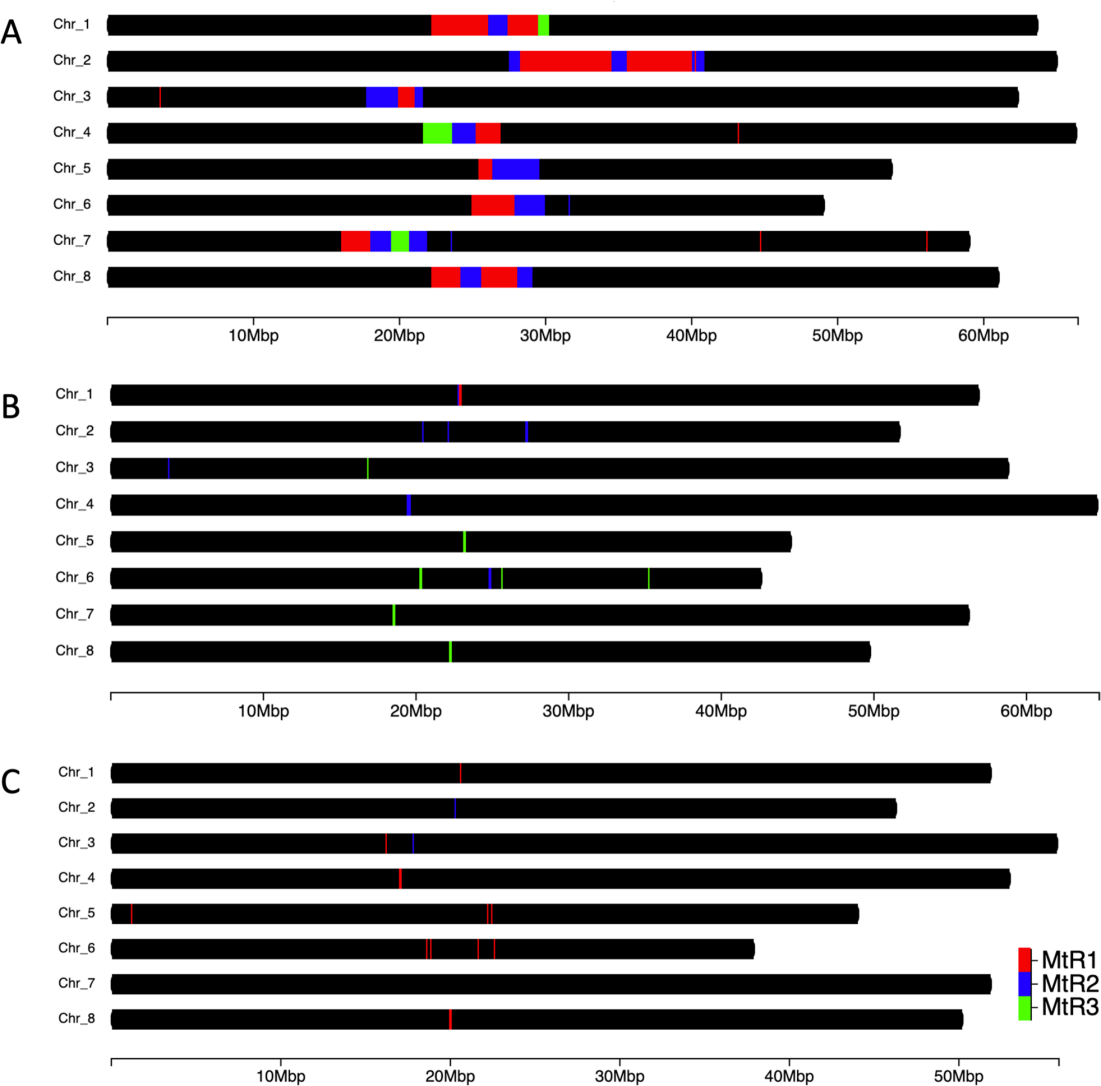
Alignment of FISH probes for pericentromeric (*Mt*R1, *Mt*R2) and centromeric (*Mt*R3) repeats of *M. truncatula*. FISH probes aligned to the (**A**) *MtHM078 v1.0* genome of HM078 (204,985 alignments), (**B**) the *Mt5.0* genome of A17 (2,354 alignments), and the (**C**) *MedtrR108_hic* genome of R108 (1,048 alignments). Overall, a much larger proportion of centromeric-related repetitive DNA was present in the *MtHM078 v1.0* genome, than observed in the reference genomes of A17 and R108

Repetitive elements were interspersed throughout the *MtHM078 v1.0* genome totaling to 218.58 Mbp, or 44.27% of the assembly size. Of this 110.73 Mbp, or 22.43% of the genome were retrotransposons of various types. In total, 92.15 Mbp, or 18.66% was composed of LTR-RTs, with the majority of those being classified as LTR Gypsy/DIRS1. Approximately 40.33 Mbp, or 8.15% of the total genome size, was classified as DNA transposons, 8.14 Mbp was small RNA elements, 4.82 Mbp was simple repeat elements, and 1.14 Mbp was low complexity repeat elements. The *MtHM078 v1.0* genome contained a similar quantities of repetitive elements as the *Mt5.0* genome. However, in the *MtHM078 v1.0* genome there was a larger proportion of unclassified repetitive elements and small RNA (Additional file 2: Table S3). Whole-genome alignments between *MtHM078 v1.0*, *Mt5.0*, and *MedtrR108_hic*, visualized with SyRI v1.6.3 [22] showed multi-megabase regions assembled in the center of HM078 pseudo-chromosomes that do not align to the reference genomes of A17 and R108 (Fig. 1c). In the *Mt5.0* assembly, numerous duplications are observed relative to the *MtHM078 v1.0* assembly, which occur in centromeric regions (Fig. 1c). A gene density plot showed that these are gene-poor regions in the *MtHM078 v1.0* assembly (Additional file 1: Figure S3). Centromeric and pericentromeric repeats were examined by the alignment of FISH probes designed for centromeric repeats of *M. truncatula* A17 [30]. The BLASTn alignments of pericentromeric, *Mt*R1 and *Mt*R2, and centromeric, *Mt*R3, FISH probes resulted in 204,985 alignments to the *MtHM078 v1.0* genome, 2,354 alignments to the *Mt5.0* genome, and 1,048 alignments to the *MedtrR108_hic* genome (Fig. 2). Overall, a much larger proportion of sequences homologous to centromeric repeats were observed in the *MtHM078 v1.0* genome assembly.

Due to the concern that repetitive regions can be prone to mis-assembly, we analyzed long-read coverage across the centromeric and pericentromeric regions. After examining high-quality alignments (Q>30) of PacBio CCS HiFi long-reads (>15 kbp) to these regions on each *MtHM078 v1.0* pseudo-chromosome, we found the median long-read coverage ranged from 23-34, while mean long-read coverage ranged from 58.78-129.8. A visualization of long-read coverage across the centromeric and pericentromeric regions of the *MtHM078 v1.0* genome showed that these regions are supported by continuous high-quality sequence data (Additional file 1: Figure S4).

### Annotation of protein-coding genes and identification of QTL regions

Illumina RNA sequencing of HM078 tissues generated 972 million reads, with mean library depth of 63 million paired-end reads per sample (Q>30). Structural annotation of the *MtHM078 v1.0* genome resulted in 37,803 protein-coding genes (Table 2). The average gene model, intron, and exon length was 2.97 kbp, 0.47 kbp, and 0.26 kbp, respectively. A total of 31,145 genes, or 82.39%, had an InterPro match and were assigned gene ontology (GO) terms. Gene density evaluated over non-overlapping 1 Mbp windows ranged from 0-142 genes/Mbp, with a genome-wide average of 81.23 genes/Mbp across windows with genes present (Additional file 1: Figure S3). A total of 98.4% of BUSCO proteins were annotated and complete. Functional annotations of the *MtHM078 v1.0* predicted proteins resulted in a total of 664 putative disease resistance genes (Additional file 2: Table S4). These were annotated as TIR-NBS-LRR genes (*n*=324), LRR and NB-ARC domain disease resistance proteins (*n*=106), NB-ARC domain disease resistance protein (*n*=93), NBS-LRR disease resistance protein (*n*=72), and CC-NBS-LRR genes (*n*=61). TIR-NBS-LRR genes were visualized across the chromosome-level genomes of A17, HM078, and R108, which revealed a high degree of clustering occurring in syntenic regions between the accessions (Fig. 1c). Additionally, 2,340 transcription factor genes were annotated. Members of the top ten most abundant families included C2H2 (*n*=305), MADS-box (*n*=107), WRKY (*n*=106), WD40-like (*n*=186), MYB (*n*=162), bHLH (*n*=85), AP2 (*n*=76), NAC (*n*=73), BZIP (*n*=69), and B3-domain transcription factor families (*n*=63) (Additional file 1: Figure S5, Additional file 2: Table S6).

**Table 2:** Genome annotation metrics for the *MtHM078 v1.0*, *Mt5.0*, and *MedtrR108_hic* genomes

	***MtHM078 v1.0* (HM078)**	***Mt5.0* (A17)**	***MedtrR108_hic* (R108)**
Protein-coding gene models	37,803	44,623	39,027
Average protein length (amino acids)	410.29	338.23	366.69
Average protein-coding gene model length (bp)	2,974.30	2,963.65	3,032.92
Average intron length (bp)	476.6	565	426.58
Average exon length (bp)	265.4	342	252.4
BUSCO complete fabales dataset (proteins)	C:98.4% [S:95.6%, D:2.8%], F:0.4%, M:1.2%, n:5366	C:97.4%[S:94.7%,D:2.7%],F:1.2%,M:1.4%,n:5366	C:97.2%[S:94.0%,D:3.2%],F:0.4%,M:2.4%,n:5366

BUSCO abbreviations are *C* Complete, *S* Complete and single-copy, *D* Complete and duplicated, *F* Fragmented, *M* Missing, *N* total BUSCOs analyzed

The QTL *rnpm1* was located on chromosome 4 between 7.5-8.5 Mbp in *MtHM078 v1.*0 with 92 annotated genes, 6.5-7.5 Mbp in *Mt5.0* with 113 annotated genes, and 6.3-7.3 Mbp in *MedtrR108_hic* with 101 annotated genes (Additional file 2: Table S8). Marker “AW256637” was identified by Kamphuis et al. [12] as tightly linked to QTL *rnpm1* in an F_2_ population. We found that this marker lies in the first coding region of a TIR-NBS-LRR gene MtrunA17_Chr4g0009041 in A17, which was homologous to medtr.HM078.gnm1.ann1.g15645. In *MtHM078 v1.*0, a cluster of ten TIR-NBS-LRR genes were identified in *rnpm1* with pairwise amino acid sequence identities ranging from 42-99.2% compared to putative homologs in *Mt5.0* (Additional file 2: Table S5). Furthermore, MYB, MADS-box, C2H2, and B3-domain transcription factors were identified in *rnpm1*, with pairwise amino acid sequence identities of 73-100% between putative homologs of A17 and HM078 (Additional file 2: Table S7).

The QTL *rnpm2* was identified by flanking markers on chromosome 4 from 55.9-56.4 Mbp in *Mt5.0* with 63 annotated genes, and is consistent with a length of 0.8 cM described by Kamphuis et al. [12]. In *Mt5.0*, the left flanking marker “h2_16a6a” lies in an intronic region of MtrunA17_Chr4g0064501, a putative sucrose-phosphate synthase, and the right flanking marker “h2_21h11d” resides in the first CDS of MtrunA17_Chr4g0065151, a putative protein-synthesizing GTPase. Due to the chromosome 4-8 translocation, *rnpm2* was located on chromosome 8 in the *MedtrR108_hic* and *MtHM078 v1.0* genomes. Specifically, *rnpm2* extended from 41.67-42.12 Mbp in *MedtrR108_hic* with 51 annotated genes, and 51.99-52.48 Mbp in *MtHM078 v1.0* with 54 annotated genes (Additional file 2: Table S8). While, no plant disease resistance genes were annotated in *rnpm2*, a leucine-rich repeat domain superfamily gene MtrunA17_Chr4g0064941 was present. Finally, only one transcription factor, a C2H2 family, was identified in *rnpm2*, with pairwise amino acid sequence identities of 99.6% between A17 and HM078 homologs (Additional file 2: Table S7).

### Analysis of indels across QTL *rnpm1 *and *rnpm2* identifies promising candidate genes

We identified indels across *rnpm1* between *Mt5.0* and *MtHM078 v1.*0 (Additional file 2: Table S9). Disruptive indels causing loss-of-function events in HM078 provide interesting candidate genes for SBS disease resistance that fit the recessive inheritance model. Six insertions and six deletions were found across *rnpm1*, and one highly diverged region. Notably, the HM078 homolog of a glutathione S-transferase (MtrunA17_Chr4g0008781) contained a 12 bp insertion in the 5’ untranslated region (UTR). Moreover, a B3-domain transcription factor (MtrunA17_Chr4g0009491) contained two frameshift variants that result in a 20 amino acid insertion and a 98 amino acid deletion in the HM078 homolog. Multiple plant disease resistance genes contained disruptive indels (Additional file 2: Table S5). For instance, a single base pair insertion in the coding region of TIR-NBS-LRR (MtrunA17_Chr4g0009401) resulted in a frame-shift mutation in the HM078 homolog (medtr.HM078.gnm1.ann1.g15660) that produced a downstream premature stop codon. This truncation of medtr.HM078.gnm1.ann1.g15660 likely causes a loss-of-function due to the absence of the LRR domain in the predicted peptide. Interestingly, a homologous sequence for this TIR-NBS-LRR (MtrunA17_Chr4g0009401) was identified in the *MedtrR108_hic* genome of SBS-susceptible accession R108, but was absent from the genome of SBS-resistant accession SA28064 (HM002) based on BLASTn alignments. However, the Illumina-based assembly of HM002 is not as complete as the PacBio-based assembly of R108, and further validation is this presence-absence variation in HM002 is needed.

In our analysis of indels in *rnpm1* we found a highly diverged region (HDR6128), which represents a 10.85 kbp insertion in the second coding sequence of *PHO2A* (MtrunA17_Chr4g0009054), which causes a loss-of-function in HM078 (Additional file 2: Table S9). This large insertion is composed of repetitive sequence that has homology to Ty3/gypsy LTR-RT based on the PlantRep repeat database Luo et al. [29]. However, this LTR-RT-like insertion only contains two ribonuclease H domains, and lacks other requisite domains for LTR-RT functionality. BLASTn searches of the *MtHM078 v1.0* genome and PacBio DNA sequence reads of HM078 support the *PHO2A* gene has been disrupted by this insertion and that *PHO2A* was not present elsewhere in the genome, which was further validated by PCR (Additional file 1: Figure S6).

Next, indels across *rnpm2* between *Mt5.0* and *MtHM078 v1.*0 were identified (Additional file 2: Table S9). In *rnpm2*, three deletions disrupting coding regions of genes were found. First, a two base pair deletion in a glycosidase (MtrunA17_Chr4g0064811) resulted a premature stop codon and loss-of-function, with no homologous gene being annotated in the *MtHM078 v1.*0 genome. Second, a single base pair deletion resulting in a premature stop codon in the F-box family gene (MtrunA17_Chr4g0065061) generated a 15 amino acid truncation in the HM078 homolog (medtr.HM078.gnm1.ann1.g36341). The same truncation was observed in a putative homologous sequence within the genome of SBS-resistant accession SA28064 (HM002) accessed from LIS (Additional file 2: Table S1). However, further validation of this is required because the HM002 genome is currently unannotated. The third deletion occurred in hypothetical protein (MtrunA17_Chr4g0065121), which resulted in no homologous gene being annotated in the *MtHM078 v1.*0 genome (Additional file 2: Table S9).

### Genes identified in the missense SNP analysis overlap with previously identified candidates

Missense SNPs were compared for genes in *rnpm1* and *rnpm2* to identify missense SNPs that were present in HM078 and absent from A17. In *rnpm1*, a total of 64 missense SNPs were found in 26 genes, and in *rnpm2* there were 19 missense SNPs in 13 genes (Additional file 2: Table S10). Some candidate genes previously identified during the indel analysis overlapped with candidate genes identified in the missense SNP analysis. In *rnpm1*, a cluster of four neighboring TIR-NBS-NLR genes (MtrunA17_Chr4g0009041-MtrunA17_Chr4g0009401) all contained 1-5 missense SNPs. The TIR-NBS-NLR gene MtrunA17_Chr4g0009041 contains the marker “AW256637” linked to *rnpm1* segregation, and was found to have two missense SNPs (freebayes-var-4-7079659-A, freebayes-var-4-7079919-T) present in the HM078 homolog medtr.HM078.gnm1.ann1.g15645. The TIR-NBS-NLR gene MtrunA17_Chr4g0009401 was previously identified as having a disruptive frameshift mutation during the indel analysis also contains a missense SNP (freebayes-var-4-7332043-C). In *rnpm2*, an F-box family gene (MtrunA17_Chr4g0065061) previously found to have a frameshift mutation in the indel analysis, was also found to have three missense mutations (freebayes-var-4-56276390-A, freebayes-var-4-56276670-A, freebayes-var-4-56276783-T) present in HM078 and absent from A17. Interestingly, the missense variant freebayes-var-4-56276390-A occurred in the F-box associated domain of this gene, which was determined using SMART [46], while the other two occurred outside of annotated domains. The top homologs for this F-box family gene in *A. thaliana* TAIR10 include F-box family protein At3g06240 (27.9% pairwise amino acid sequence identity), and two splice variants of F-box family protein At4g12560 (23.1% pairwise amino acid sequence identity) also known as *CPR1/CPR30* (constitutive expressor of pathogenesis-related genes 1 and 30). In summary, F-box family gene (MtrunA17_Chr4g0065061) was identified as a strong candidate gene for SBS-disease resistance based on multiple structural variants in the HM078 homolog medtr.HM078.gnm1.ann1.g36341.

### Micro-synteny viewer shows structural variation associated with candidate genes

**Fig 3:**
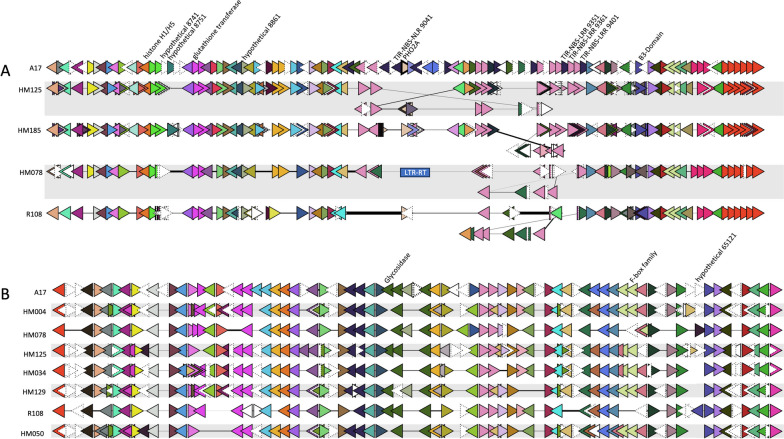
Micro-synteny of QTL regions *rnpm1* and *rnpm2* for selected *M. truncatula* genomes (**A**) The center of QTL region *rnpm1* (~400 kbp) with candidate genes for SBS disease resistance labeled. Genes displayed in pink and dark purple represent TIR-NBS-LRR plant disease resistance genes and appear to be associated with local rearrangements. The LTR-RT-like repeat sequence which disrupted the *PHO2A* gene in HM078 is labeled with a blue box. **B** The entire QTL region *rnpm2* (~480 kbp) with candidate genes for SBS disease resistance labeled. F-box family genes are represented by light green and mauve triangles*.* Triangles represent annotated genes, which are colored based on legume federation gene family assignments within a single plot

Micro-synteny across *rnpm1* and *rnpm2* regions was examined for annotated genomes of *M. truncatula* available on LIS, and the *MtHM078 v1.0* genome. Candidate genes for SBS disease resistance were identified based on indels, missense SNPs, marker presence, or PAV and labeled in GCV plots (Table 3, Fig. 3). Interestingly, we observed structural variation that was associated with several candidate genes identified in the indel and missense SNP analysis.

**Table 3:** Candidate genes for SBS disease resistance in QTL regions *rnpm1* and *rnpm2*

**QTL**	**Gene (A17 5.0)**	**Gene (HM078)**	**Rationale for selection**	**Description (A17)**	**EMBOSS Needle Alignment Identity (amino acid)**
*rnpm1*	MtrunA17_Chr4g0008711	medtr.HM078.gnm1.ann1.g15616	Indel	Linker histone H1/H5	128/176 (72.7%)
	MtrunA17_Chr4g0008741	*NA*	Indel	Hypothetical protein	*NA*
	MtrunA17_Chr4g0008751	*NA*	Indel	Hypothetical protein	*NA*
	MtrunA17_Chr4g0008781	medtr.HM078.gnm1.ann1.g15622	Indel	Glutathione S-transferase	216/220 (98.2%)
	MtrunA17_Chr4g0008861	medtr.HM078.gnm1.ann1.g15629	Missense SNP & Indel	Hypothetical protein	925/943 (98.1%)
	MtrunA17_Chr4g0009041	medtr.HM078.gnm1.ann1.g15645	Missense SNP & contains marker	TIR-NBS-LRR	1482/1720 (86.2%)
	MtrunA17_Chr4g0009054	*NA*	Indel/PAV	Ubiquitin-conjugating enzyme E2 (*PHO2A*)	*NA*
	MtrunA17_Chr4g0009351	*NA*	Indel	TIR-NBS-LRR	*NA*
	MtrunA17_Chr4g0009361	medtr.HM078.gnm1.ann1.g15657	Indel	TIR-NBS-LRR	365/698 (52.3%)
	MtrunA17_Chr4g0009401	medtr.HM078.gnm1.ann1.g15660, medtr.HM078.gnm1.ann1.g15661	Missense SNP & Indel	TIR-NBS-LRR	702/1673 (42.0%), 937/1673 (56.0%)
	MtrunA17_Chr4g0009491	medtr.HM078.gnm1.ann1.g15670	Indel	Transcription factor B3-Domain family	330/452 (73.0%)
*rnpm2*	MtrunA17_Chr4g0064811	*NA*	Indel	Glycosidase	*NA*
	MtrunA17_Chr4g0065061	medtr.HM078.gnm1.ann1.g36341	Missense SNP & Indel	F-box domain, F-box associated interaction	361/380 (95.0%)
	MtrunA17_Chr4g0065121	*NA*	Indel	Hypothetical protein	*NA*

For instance, in *rnpm1* micro-rearrangements were observed surrounding the *PHO2A* locus. This locus is flanked on both sides by multiple TIR-NBS-LRRs, which also include the candidate TIR-NBS-LRR gene MtrunA17_Chr4g0009401. Gene family order across *rnpm1* is largely conserved between *M. truncatula* accessions, except for the genes occurring within the TIR-NBS-NLR cluster, which contains approximately ten genes upstream and thirty genes downstream of *PHO2A* (Fig. 3). Micro-synteny of QTL *rnpm2* was examined for annotated genomes of *M. truncatula*. Overall, gene family order across *rnpm2* is conserved, with minor differences between accessions.

## Discussion

### Deep PacBio sequencing enables a highly contiguous assembly and near-complete annotation

The fragmentation of short-read assemblies confounds the examination of large genomic regions, such as previously described QTL for various agronomic traits that can be as large as 20 cM [66, 67]. The *Mt4.0* genome of *M. truncatula* accession A17 used Illumina short-reads assembled with ALLPATHS-LG, which resulted in a 390 Mbp assembly containing 8,026 contigs with a contig N50 of 339.06 kbp [1]. Furthermore, 15 *M. truncatula* accessions assembled with Illumina short-reads for a pan-genome project resulted in assemblies with over 3,000 contigs each and contig N50s around 20 kbp [4]. The advent of long-read DNA sequencing complemented by improvements in assembly algorithms and super-computing accessibility have permitted the generation of near-complete chromosome-level plant genomes. For instance, HiFiasm assembly of PacBio CCS HiFi long-reads has produced near chromosome-level plant genome assemblies [68, 69]. The current reference-quality chromosome-level genomes of *M. truncatula* are of accessions A17 and R108, which are both susceptible to SBS disease. Previously, A17 (HM101) has been used as a susceptible genotype and SA27063 (HM078) as a resistant genotype for studying SBS disease [12, 70]. We applied 142x coverage of PacBio HiFi CCS long-read DNA sequencing to produce a highly contiguous genome of *M. truncatula* HM078 to provide a resource for studying SBS disease resistance. After genome annotation using the BRAKER/TSEBRA pipeline, there were 37,803 protein coding genes and 35,845 (94.8%) were homologous to conserved legume gene families. BUSCO analysis of the *MtHM078 v1.0* genome revealed that 99% of universal fabales orthologs were present and 2.8% were duplicated.

The majority of missing BUSCOs from the *MtHM078 v1.0* genome were also missing from the *Mt5.0* and *MedtrR108_hic* genomes. This suggests that the BUSCO fabales dataset may contain orthologs that are not present in *M. truncatula*, or are present in genomic regions that are recalcitrant to genome assembly using the current methods. After genome annotation, BUSCO analysis of the *MtHM078 v1.0* predicted proteins found that 98.4% of the universal fabales orthologs were present, which supports a near-complete haploid assembly with limited duplication.

### Comparison of *M. truncatula *chromosome-scale genomes reveals large proportion of centromeric-related repeats in the *MtHM078 v1.0* genome

Recently, RagTag has been used to orient contigs into chromosome-level plant genomes for tomato, strawberry, and mangrove based on reference genomes [71–73]. For instance, contigs of the *C. zippeliana* (mangrove) genome were scaffolded based on the chromosome-level assembly of a related species *C. tagal* [72]. In this study, RagTag v2.1.0 [74] was utilized to orient HM078 contigs to chromosomes 1-8 of the *MedtrR108_hic* genome because R108 lacks the interchromosomal translocation found in A17 [13]. Scaffolding of the *MtHM078 v1.0* genome resulted in a 481.19 Mbp assembly size, with chromosome sizes that were 2.63-25.44% and 11.66-39.95% larger than the *Mt5.0* and *MedtrR108_hic* genome, respectively. Based on c-values, the haploid genome size of *M. truncatula* is 465 Mb, meaning that the *MtHM078 v1.0* genome is 3.48% larger than the estimated genome size. Whole-genome pairwise alignments between the chromosome-level assemblies of HM078, A17, and R108 revealed multi-megabase regions in the center of HM078 pseudo-chromosomes, which are not seen to the same extent in the reference genomes for A17 and R108. Alignment of FISH probes, designed for pericentromeric and centromeric repeats of A17 revealed that these regions are composed of centromeric-related repetitive DNA in the *MtHM078 v1.0* genome [30]. Interestingly, when we aligned these probes to the *Mt5.0* and *MedtrR108_hic* genome we found the *MtHM078 v1.0* genome contains 87 and 195 times more BLASTn alignments (sequence identity >98%, e-value <1e-05), respectively. From a biological viewpoint, these three accessions likely have similar centromeres sizes, and the differences we observed in the assemblies are a function of using higher genome-wide coverage of long-read sequence data which can generate more complete assemblies of repetitive regions [68]. Overall, the *MtHM078 v1.0* genome offers a novel sequence resource for studying centromeric repeats in *M. truncatula*.

### Newly discovered candidate genes for SBS disease resistance

We utilized genomic resources of *M. truncatula*, cutting-edge sequencing technologies, and recently developed software to produce a high-quality genome assembly of SBS-resistant HM078, which enabled a sequence-level examination of two QTL regions reported by Kamphuis et al. [12]. Candidate genes were identified in each region based on structural variation, such as indels or missense SNPs, that result in potential loss-of-function events or amino acid differences in HM078 homologs. Indels were determined based on SyRI v1.6.3 pairwise alignment between SBS-susceptible A17 and SBS-resistant HM078. The indel analysis identified multiple candidate genes, whose biological function suggests a role in SBS disease resistance. Finally, missense SNPs were evaluated between A17 and HM078 to identify HM078 homologs across *rnpm1* and *rnpm2* with different amino acid predictions. Based on our analysis of structural variants in these QTL regions we found several candidate genes whose function aligned with a biological role in disease resistance to necrotrophic fungi.

Our analysis of indels affecting genes in *rnpm1* identified a glutathione S-transferase (GST) and a B3-domain transcription factor as candidate genes for SBS disease resistance. Previous research has shown that GSTs participate in detoxification of xenobiotic toxins, reduction of oxidative stress, and are receptors of salicylic acid [75]. In HM078, the GST medtr.HM078.gnm1.ann1.g15622 contained a 12 bp insertion in the 5’ UTR region, which is known to contain microRNA binding sites and structural features that regulate mRNA splicing and stability [76]. For the B3-domain transcription factor candidate gene, a single base pair deletion and a two base pair insertion in the HM078 homolog caused a 98 amino acid deletion and truncation of a B3 domain. A recent study in *Populus* spp. revealed that B3-domain transcription factors are co-expressed during secondary cell walls biosynthesis and lignin formation [77]. Based on what is known about GSTs and B3-domain transcription factors, they could participate in the host response to necrotrophic fungal pathogens.

Some necrotrophic fungal pathogens have adapted to exploit disease resistance proteins. For instance, a plant disease resistance protein LOCUS ORCHESTRATING VICTORIN EFFECTS1 (*LOV1*) in oat confers sensitivity to the HST victorin produced by *C. victoriae* [78]. This phenomenon has been described as the inverse gene-for-gene model, where a HST is recognized by a dominant host gene resulting in effector-triggered susceptibility (ETS) [79]. Another example, *LAZ5*, is a TIR-NBS-LRR protein, where a loss-of-function confers resistance to *S. sclerotiorum* [61]. Kamphuis et al. [12] identified two recessively inherited QTL, *rnpm1* and *rnpm2*, which were associated with SBS disease resistance in *M. truncatula*. We examined a cluster of TIR-NBS-LRR genes in *rnpm1*, and identified candidates, including MtrunA17_Chr4g0009401, where a frameshift resulted in a downstream premature stop codon in the HM078 homolog. Based on what is known regarding necrotrophic susceptibility and the inverse gene-for-gene model, a loss-of-function in a TIR-NBS-LRR gene, could alleviate sensitivity to a HST that has yet to be identified.

The *PHOSPHATE2* (*PHO2*) paralogs are functionally annotated as ubiquitin conjugating E2 enzymes that contribute to P_i_ homeostasis in plants. A loss-of-function in *PHO2* paralogs has been shown to result in hyper-accumulation of P_i_ in *M. sativa* and *M. truncatula* [80, 81]. In *A. thaliana*, wild-type plants grown in high P_i_, *pho2* loss-of-function mutants, and over-expression of miR399 all result in higher P_i_ concentrations in their shoots, which are accompanied by the accumulation of reactive oxygen species (ROS), increased JA and SA levels, and an upregulation of dependent defense genes [82]. Overall, *pho2* mutants showed increased resistance to necrotrophic fungal pathogen *P. cucumerina.* Interestingly, our analysis of indels affecting genes in *rnpm1* enabled the identification of a loss-of-function of *PHO2A* in HM078 due to a 10.85 kbp insertion of an LTR-RT-like sequence. However, in *M. truncatula*, *PHO2A* was shown to have very low expression compared to the *PHO2B* and *PHO2C* paralogs, which also have roles in symbiotic nitrogen fixation and P_i_ homeostasis. For nodulated plants, *PHO2A* had the highest expression in shoot tissue during phosphate starvation [80]. Based on previous research, the hyperaccumulation of P_i_ in *M. truncatula* may contribute to disease resistance against necrotrophic fungal pathogens, potentially due to the effect of elevated P_i_ on defense gene pathways.

Our analysis of QTL *rnpm2* revealed this region has a high degree of synteny across annotated *M. truncatula* genomes. Interestingly, an F-box protein interaction domain protein (medtr.HM078.gnm1.ann1.g36341) in *rnpm2* contained several missense mutations as well as a frameshift variant causing a truncation in HM078. A missense mutation was found in the F-box associated domain, which is involved in ubiquitination of target proteins for degradation by the proteosome. The predicted protein was similar to the *A. thaliana* F-box family gene *CPR1/CPR30*, which has been shown to be a negative regulator of TIR-NBS-LRR protein SNC1 (Suppressor of npr1-1 constitutive 1). The *cpr1/cpr30* mutants exhibited constitutive *PR1/PR2* defense responses and dwarfism dependent on EDS1 (Enhanced disease susceptibility 1) and PAD4 (Phytoalexin deficient 4), which are required for SA defense response signaling [83]. A missense mutation in *SNC1* was found to cause the activation of constitutive defense responses [84]. Based on previous research, this promising candidate gene could be acting as a negative regulator of unidentified plant disease resistance proteins.

Investigating the interactions between *M. truncatula* and *A. medicaginicola* has the potential to reveal unknown factors that contribute to SBS disease resistance, and ultimately improve the agronomic performance of legumes. We leveraged advances in sequencing technologies to produce a high-quality genome assembly of *M. truncatula* accession SA27063 (HM078) to provide a resource for the investigating SBS disease resistance. Amazingly, only twenty-three contigs made up 481.19 Mbp, which were oriented to build HM078 pseudo-chromosomes. The genome and predicted proteins were both near-complete, containing as many BUSCO genes as the other reference-quality genomes of *M. truncatula*. Eleven and three candidate genes were identified across QTL regions *rnpm1* and *rnpm2*, respectively. Future research will include the generation CRISPR-Cas-mediated homozygous mutants in *M. truncatula* followed by SBS disease screening for promising candidate genes such a TIR-NBS-LRR and *PHO2A* in *rnpm1*, as well as the glycosidase and F-box family gene in *rnpm2*. Upon validation of resistance genes in whole-plant mutants, prospective studies will include exploring the applicability of our findings to the agronomically important forage crop alfalfa.

### Supplementary Information

**Supplementary Material 1.**

**Supplementary Material 2.**

## Data Availability

The HM078 genome assembly has been deposited on LIS https://data.legumeinfo.org/Medicago/truncatula/genomes/HM078.gnm1.Q3TM/ along with the associated genome annotation files https://data.legumeinfo.org/Medicago/truncatula/annotations/HM078.gnm1.ann1.3RNP/ All raw sequence data has been deposited in the NCBI database under BioProject PRJNA975868. SRA number SRR24726732 contains the PacBio HiFi CCS DNA sequence reads of *M. truncatula* accession SA27063 (HM078). SRA numbers SRR24748925, SRR24748926, and SRR24748927 contain RNA-sequence reads of SA27063 (HM078) root, apical meristem, and immature leaf tissue, respectively. SRA numbers SRR24775323, SRR24775324, SRR24775325, SRR24775321, SRR24775322, SRR24793326, SRR24775317, SRR24775319, SRR24775320 contain RNA-sequence reads of mature SA27063 (HM078) leaf tissue. Associated code run throughout this study, as well as the VCF file from the genome-wide pairwise alignment between *Mt5.0* and *MtHM078* are available on GitHub https://github.com/shaun-curtin/Genome-assembly-of-Medicago-truncatula-accession-HM078. Finally, germplasm of *M. truncatula* accessions can be requested through LIS https://medicago.legumeinfo.org/tools/germplasm.

## Abbreviations

SBS: Spring black stem
QTL: Quantitative trait locus
rnpm1: Resistance to the necrotroph Phoma medicaginis one
rnpm2: Resistance to the necrotroph Phoma medicaginis two
BACs: Bacterial artificial chromosomes
WGD: Whole genome duplication
HSTs: Host-selective toxins
PTI: PAMP-triggered immunity
ETI: Effector-triggered immunity
PAMPs: Pathogen associated molecular patterns
PRR: Pattern recognition receptors
PR: Pathogenesis-related
SAR: Systemic acquired resistance
SA: Salicylic acid
JA: Jasmonic acid
ABA: Abscisic acid
ET: Ethylene
LOV1: LOCUS ORCHESTRATING VICTORIN EFFECTS1
INRA: Institut National de la Recherche Agronomique
TIR-NBS-LRR: Toll/Interleukin1 receptor-nucleotide binding site-leucine-rich repeat
CC-NBS-LRR: Coiled-coil nucleotide binding site-leucine-rich repeat
NLR: Nucleotide-binding and leucine-rich repeat
ETS: Effector-triggered susceptibility
HR: Hypersensitive response
DI: De-ionized
PDA: Potato dextrose agar
HMW: High molecular weight
PacBio: Pacific Biosciences
CCS: Circular consensus sequencing
HiFi: High fidelity
BUSCO: Benchmarking universal single-copy orthologs
TEs: Transposable elements
LINEs: Long interspersed nuclear elements
LTR-RT: Long terminal repeat retrotransposons
FISH: Fluorescence in situ hybridization
LIS: Legume Information System
GCV: Genome context viewer
HMM: Hidden Markov model
GO: Gene Ontology
PAV: Presence absence variation
CNV: Copy number variation
UTR: Untranslated region
GST: Glutathione S-transferase
CPR1: Constitutive expressor of pathogenies-related genes 1
PCD: Programmed cell death
PHO2: PHOSPHATE2
SNC1: Suppressor of npr1-1 constitutive 1
EDS1: Enhanced disease susceptibility 1
PAD4: Phytoalexin deficient 4
