# Supplementary material for: Genome assembly of Medicago truncatula accession SA27063 provides insight into spring black stem and leaf spot disease resistance
Source: BMC Genomics. 2024 Feb 23;25:204. doi: 10.1186/s12864-024-10112-9 (PMC10885650; doi:10.1186/s12864-024-10112-9)
Supplement: Supplementary file 1 — Supplementary Material 1. [file 12864_2024_10112_MOESM1_ESM.docx]

**Supplemental Figures**

**
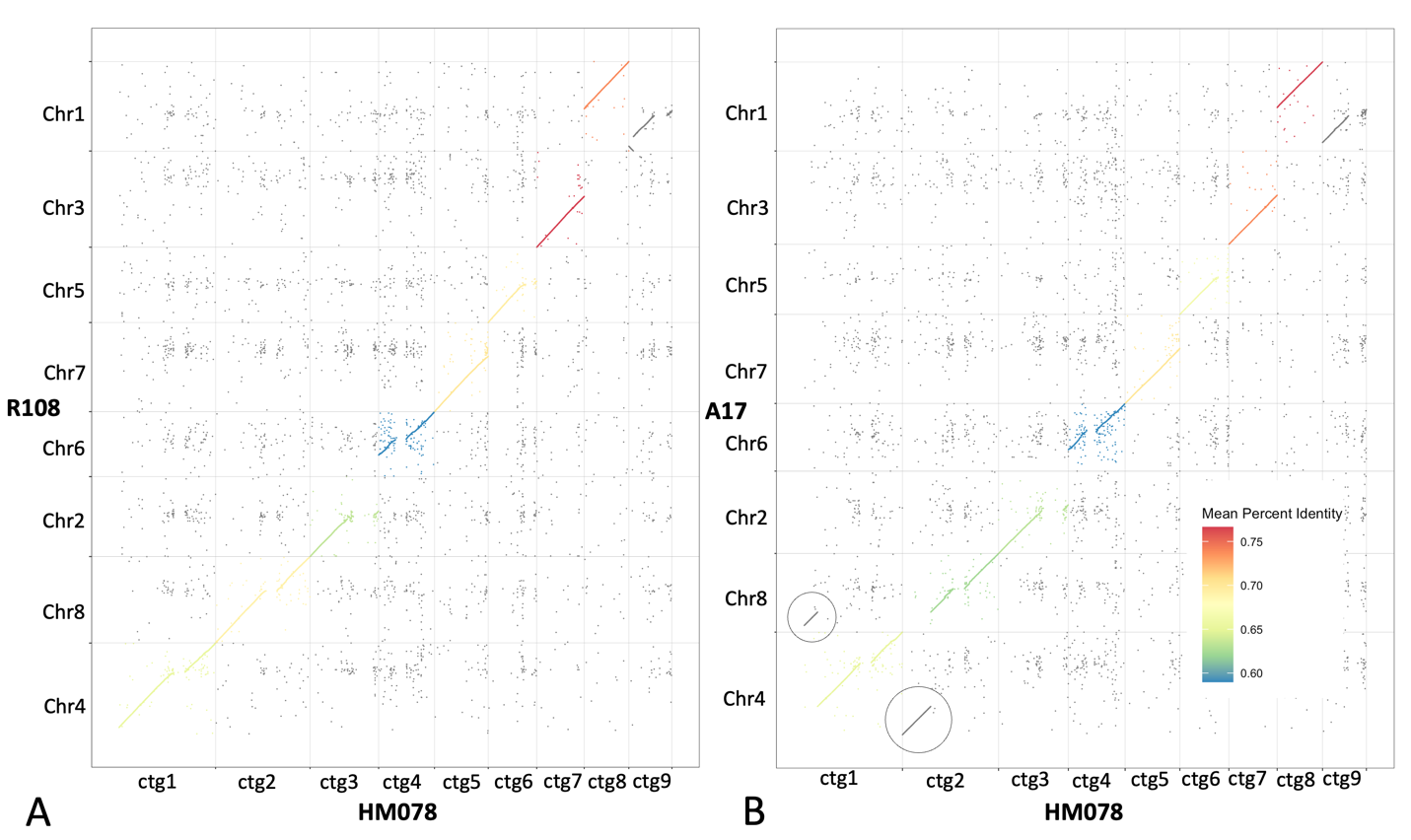
**

**Figure S1.** DNA-based alignments between chromosome-level genomes of *M. truncatula* and HM078 contigs. (A) Chromosomes of R108 aligned to the 9 largest contigs of HM078, and (B) chromosomes of A17 aligned to the 9 largest contigs of HM078. The alignment of the HM078 contigs to *M. truncatula* accessions R108 and A17 are largely similar, except for the chromosome 4-8 translocation in A17, which is not present in HM078 (circled).


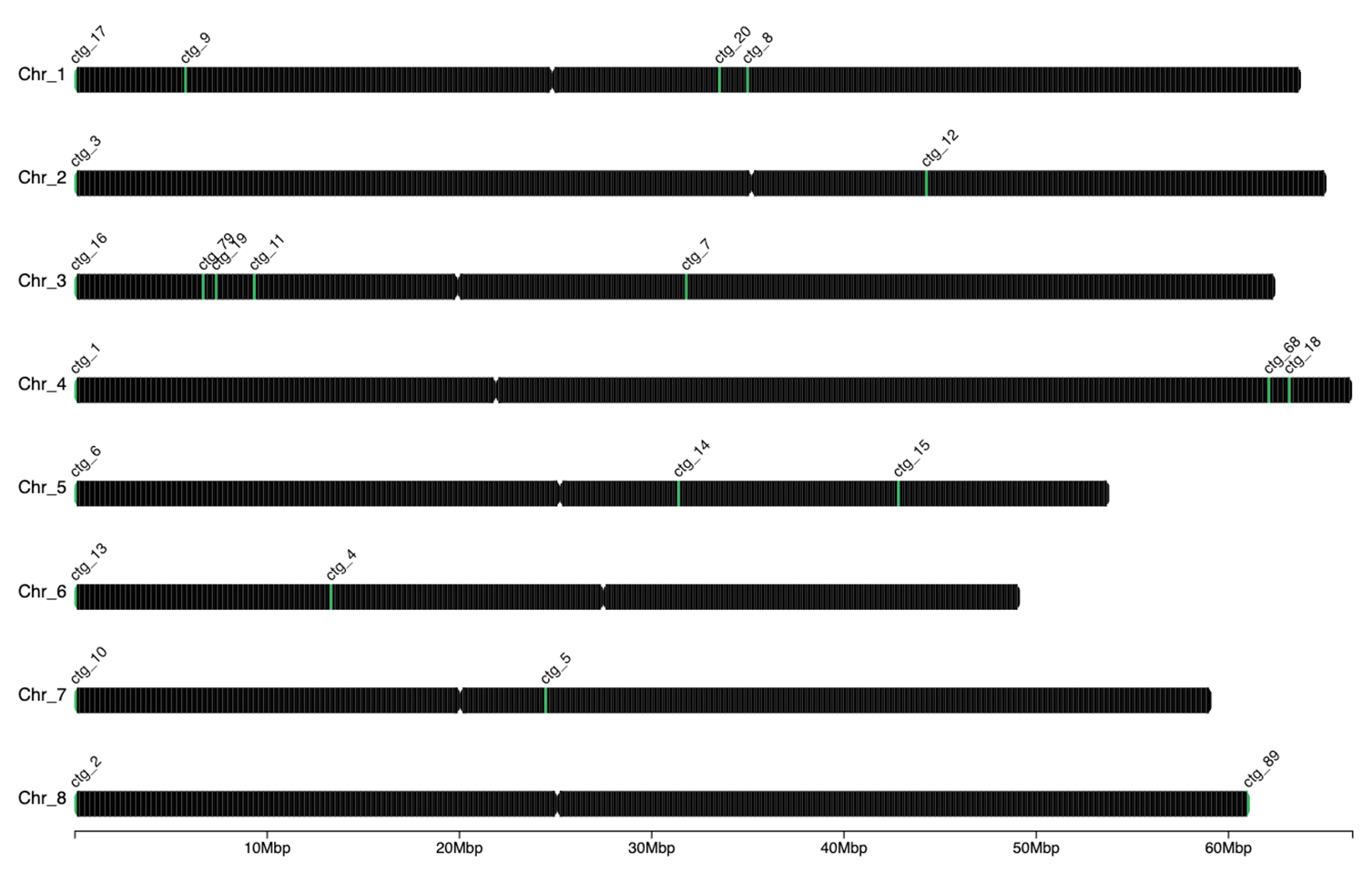


**Figure S2.** Anchoring of twenty-three *MtHM078* primary contigs to the chromosomes of the *MedtrR108_hic* genome. Start sites of *MtHM078* primary contigs are labelled in green.


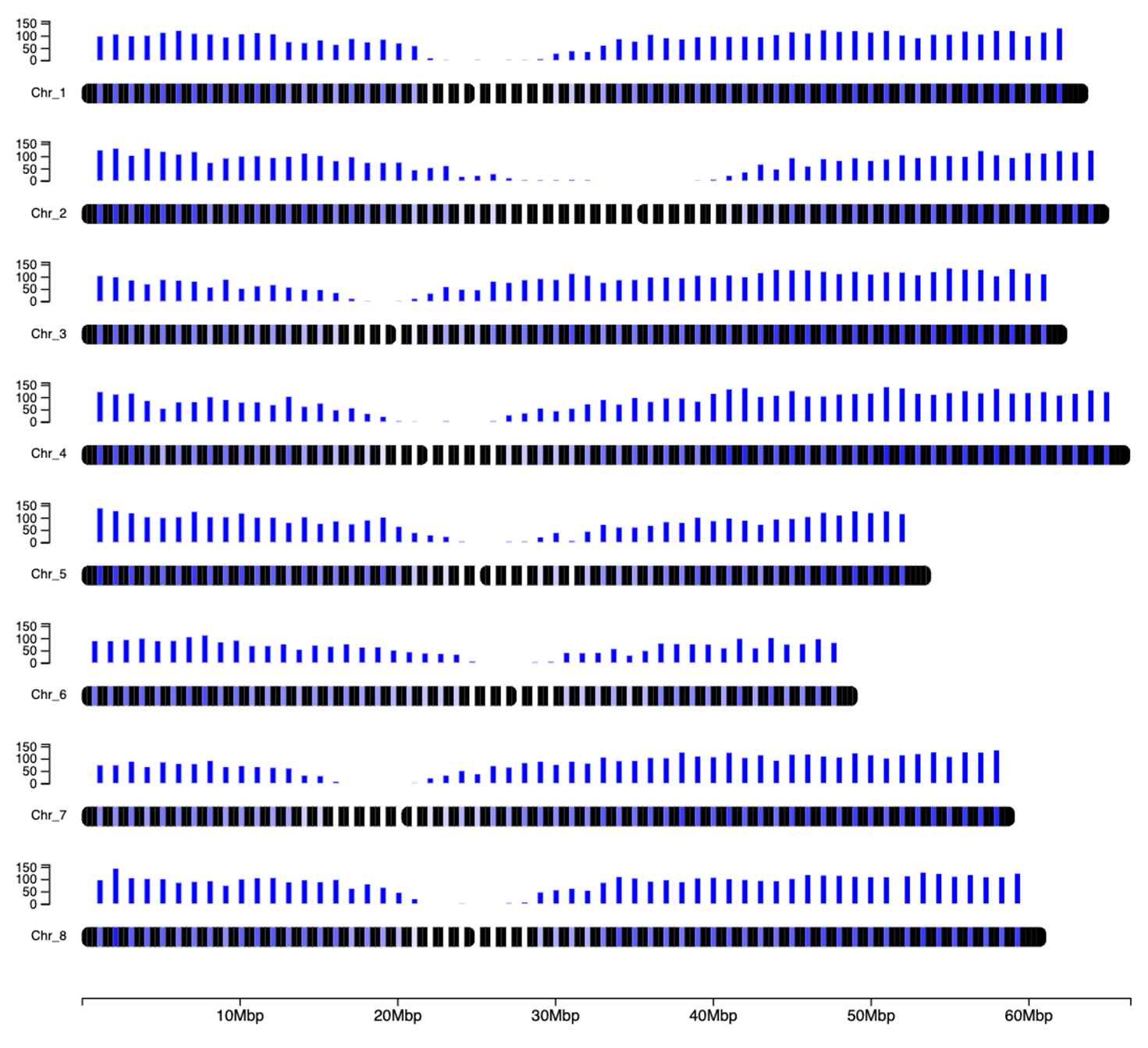


**Figure S3.** Gene density displayed across the *MtHM078 v1.0* genome. Counts, on the Y-axis, are in genes per megabase (Mbp), which are displayed by bar plots. Dark blue regions indicate high gene density, while white regions indicate no genes are annotated. Multi-megabase gene-poor regions are located approximately in the center of each pseudo-chromosome in the *MtHM078 v1.0* genome.


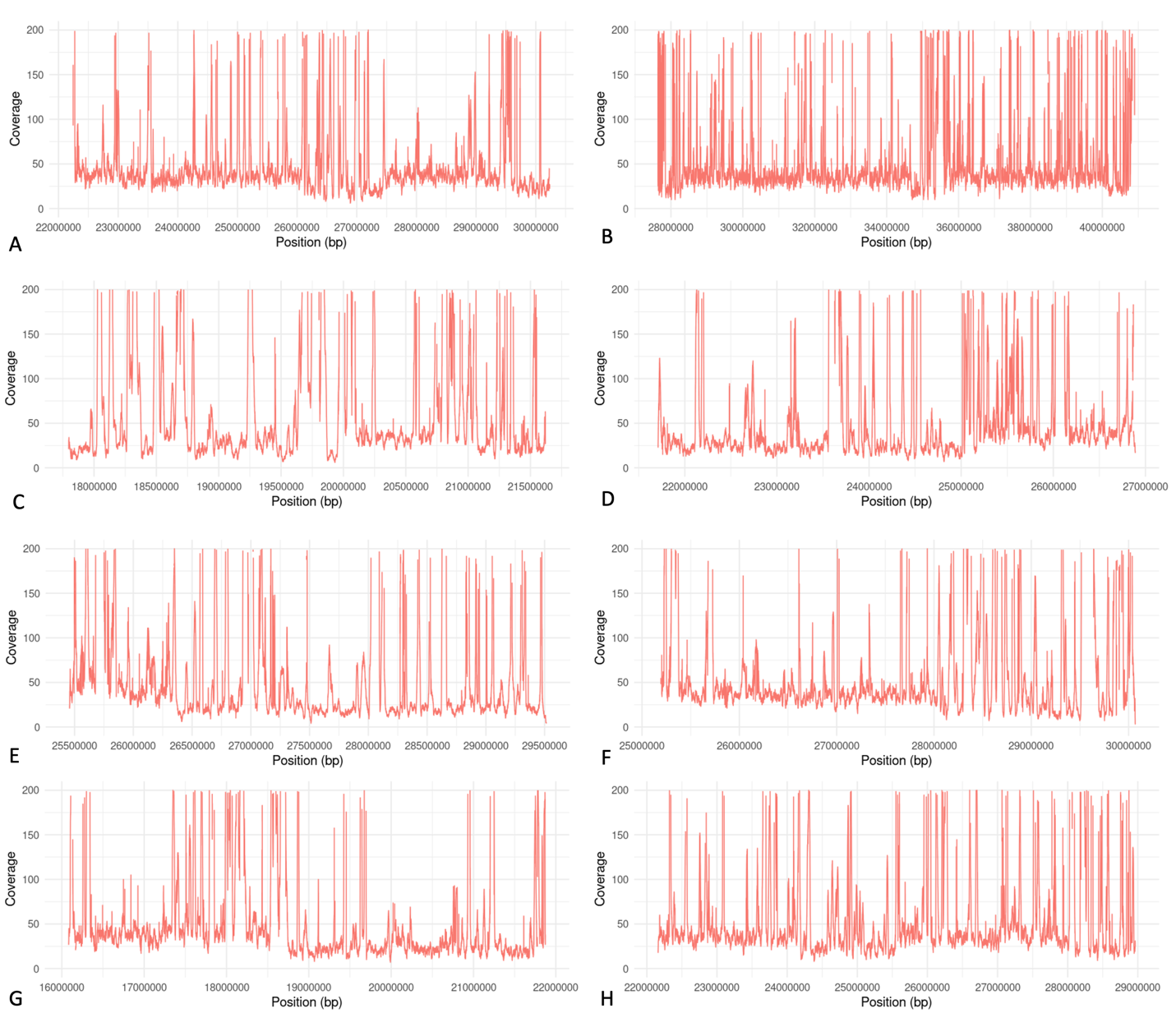


**Figure S4.** Long-read (>15 kbp) coverage of centromeric and pericentromeric regions of the *MtHM078 v1.0* genome. (A) Chromosome 1, (B) Chromosome 2, (C) Chromosome 3, (D) Chromosome 4, (E) Chromosome 5, (F) Chromosome 6, (G) Chromosome 7, (H) Chromosome 8. The median coverage of long-reads for chromosomes 1-8 ranged from 23-34, mean coverage was 58.78-129.8, minimum coverage was 3-7, and maximum coverage was 8,133-9,808. Genomic positions were homologous to centromeric and pericentromeric repeat regions.

**
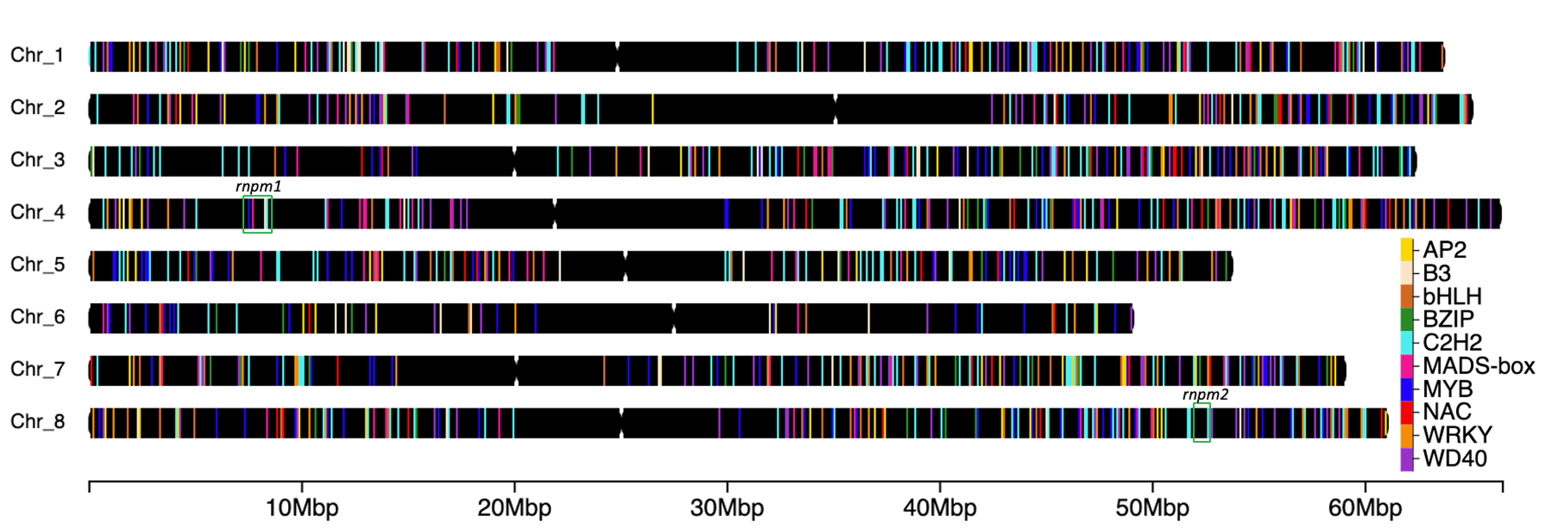
**

**Figure S5.** Transcription factor genes annotated in the *MtHM078 v1.0* genome. A total of 1,232 transcription factors are visualized genome-wide from 10 of the most abundant families including AP2, B3-domain, bHLH, BZIP, C2H2, MADS-box, MYB, NAC, WRKY, and WD40. In QTL *rnpm1*, MYB, MADS-box, C2H2, and B3-domain transcription factors were identified. In QTL rnpm2, a C2H2 transcription factor gene was identified. QTL *rnpm1* and *rnpm2* are labelled in green boxes.


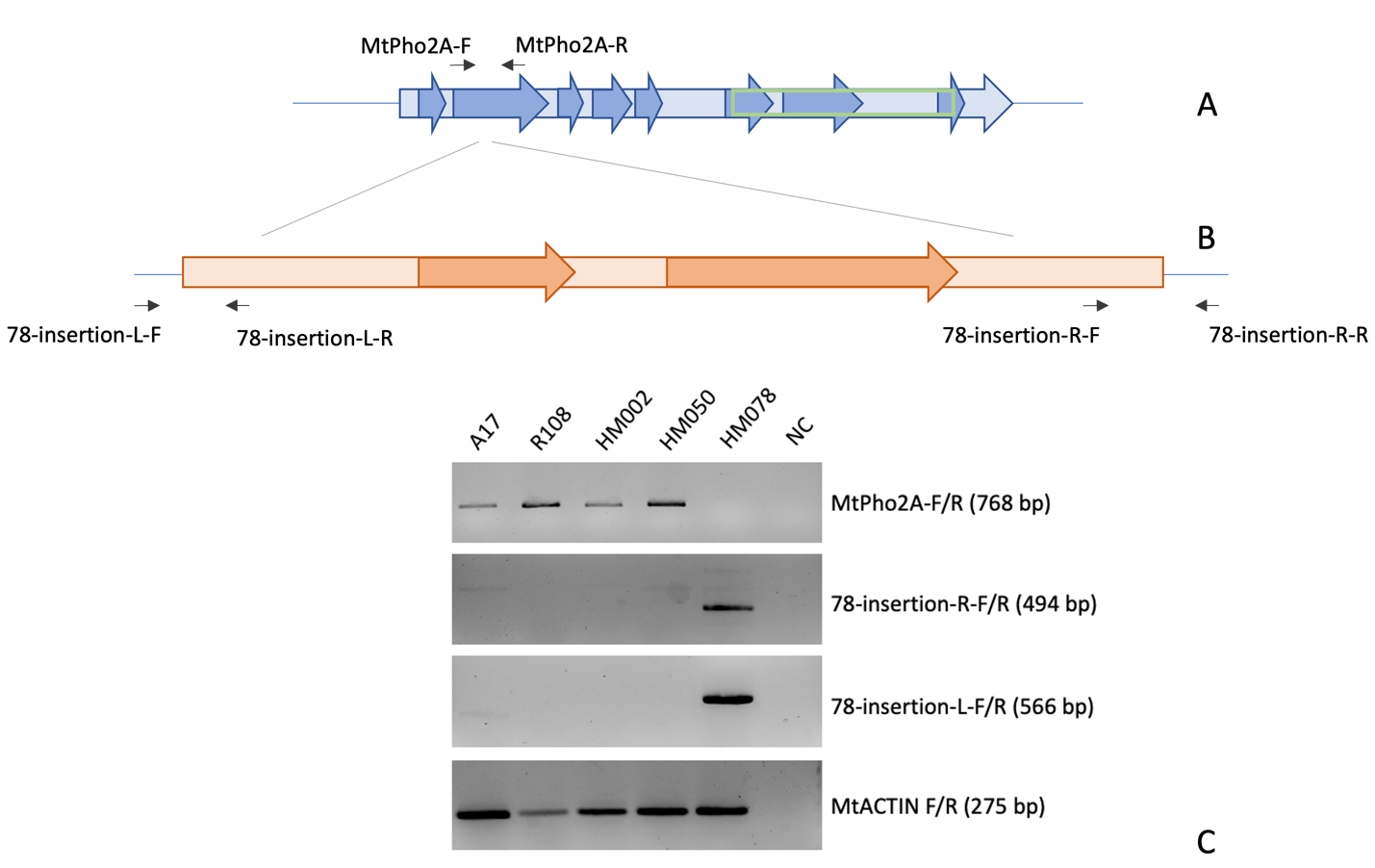


**Figure S6.** PCR amplification of the *PHO2A* locus showing LTR-RT insertion site in HM078. (A) Structure of the *PHO2A* gene and primer locations. A light blue arrow indicates the 6.4 kbp *PHO2A* gene, while dark blue arrows indicate approximate CDS locations, and the green box denotes the ubiquitin-conjugating enzyme E2 catalytic domain. (B) Structure and primer locations for an insertion of a Ty3/gypsy LTR-RT-like repeat sequence at the *PHO2A* gene locus in HM078. A light orange box indicates the 10.85 kbp LTR retrotransposon, while two dark orange arrows indicate putative Ribonuclease H domains. The LTR-RT-like repeat was inserted in the second CDS of *PHO2A*, indicated by the gray lines. (C) PCR amplification of gDNA showing the presence of the *PHO2A* locus in A17 (HM101), R108 (HM340), SA28064 (HM002), DZA058-J (HM050), and absence of the of the *PHO2A* locus in SA27063 (HM078), as well as the presence of the LTR-RT-like insertion in HM078 at the *PHO2A* locus.
